# Epidemiological Characteristics of Coxsackievirus A6 in Baotou, Inner Mongolia, China, 2023–2024

**DOI:** 10.3390/v18060680

**Published:** 2026-06-18

**Authors:** Chenxi Zhang, Yurong Yang, Rong Jin, Jiebo Xia, Hanjie Liu, Guoyong Mei, Haijun Du, Miao Jin, Zhiqiang Xia, Qinqin Song, Desheng Zhai, Jun Han

**Affiliations:** 1 National Key Laboratory of Intelligent Tracking and Forecasting for Infectious Diseases, National Institute for Viral Disease Control and Prevention, Chinese Center for Disease Control and Prevention, 155 Changbai Road, Beijing 102206, China; cimanji@outlook.com (C.Z.); 18397571843@163.com (J.X.); 18770024618@163.com (H.L.); 13439344537@163.com (G.M.); duhj@ivdc.chinacdc.cn (H.D.); jinmiao37@126.com (M.J.); xiazq@ivdc.chinacdc.cn (Z.X.); 2 School of Public Health, Henan Medical University, 601 Jin Sui Avenue, Xinxiang 453000, China; 3 Baotou Center for Disease Control and Prevention, Baotou 014000, China; yangyurong6901@126.com (Y.Y.); jinrong5171570@163.com (R.J.)

**Keywords:** HFMD, pathogen spectrum, Coxsackievirus A6, genetic evolution, amino acid variation

## Abstract

The re-emergence of Coxsackievirus A6 (CV-A6) as a predominant pathogen in hand, foot, and mouth disease (HFMD) underscores the need for ongoing molecular surveillance to clarify local evolutionary dynamics. This study aimed to characterize the genetic features of CV-A6 strains circulating in Baotou, Inner Mongolia, from 2023 to 2024. Throat swabs collected from HFMD patients were screened using real-time quantitative PCR; the VP1 region and complete genomes of representative CV-A6-positive samples were amplified and sequenced. Phylogenetic and recombination analyses were subsequently performed. Among 266 clinical specimens, 169 (63.53%) tested positive for enterovirus, of which 146 (86.39%) were identified as CV-A6. The local epidemic displayed an autumn–winter seasonality and predominantly affected children aged 4–6 years. Phylogenetic reconstruction of 133 VP1 sequences revealed that all Baotou CV-A6 isolates belonged to subgenotype D3c, and analysis of complete genomes identified a predominant recombinant form. These findings demonstrate that the D3c subgenotype, characterized by a specific recombinant structure, was responsible for HFMD outbreaks in Baotou during the study period, providing essential molecular evidence for regional public health strategies and vaccine development.

## 1. Introduction

Hand, foot, and mouth disease (HFMD) is a globally prevalent infectious disease that primarily affects children under 5 years of age [1]. Most cases are caused by *Enterovirus alphacoxsackie*, including EV-A71, CV-A16, CV-A10 and, increasingly, Coxsackievirus A6 (CV-A6) [2,3]. Additional serotypes such as CV-A2, CV-A4, CV-A5, CV-A12 and several *Enterovirus betacoxsackie* members also contribute to the disease burden [4]. Since CV-A6 was first identified as a pathogen associated with HFMD outbreaks in Finland in 2008 [5], CV-A6 has triggered successive epidemics that now span Europe, the Americas and Asia [6,7,8,9,10,11,12]. In mainland China, CV-A6-associated HFMD cases have increased markedly since 2013, with the serotype overtaking EV-A71 to become one of the predominant pathogens and thereby reshaping outbreak control strategies. Notably, CV-A6 is associated with atypical HFMD presentations; in severe cases, vesicular lesions extend beyond typical sites, with complications including onychomadesis and encephalitis [13].

Similar to other enterovirus (EV) genomes, the CV-A6 genome consists of a non-segmented, single-stranded, positive-sense RNA with a length of approximately 7400 bp [14,15,16]. The genomes of most EV genotypes contain a complete Open Reading Frame (ORF). The viral RNA functions as mRNA and is translated into a large polyprotein of approximately 2200 amino acids. This polyprotein is subsequently cleaved by viral proteases into three precursor polyproteins P1, P2, and P3. The P1 polyprotein is cleaved into capsid proteins (VP1-VP4), while the P2 and P3 polyproteins are cleaved into non-structural proteins (2A-2C, 3A-3D) [17]. Among these, VP1 is a major serotype-specific protein widely used for virus identification and evolutionary research. Based on the 15% nucleotide (NT) sequence difference threshold within the VP1 region, CV-A6 was divided into six genotypes, namely A, B (B1-B2), C (C1-C2), D (D1-D3), E and F. Among these, genotypes A, B, and C have rarely been reported in recent years. Genotype A includes the prototype strain Gdula (isolated in the US in 1949), and China’s first reported CV-A6 strain (SD/CHN/1992/JQ364886), which together formed a distinct clade. Genotype C comprises two subgenotypes (C1 and C2). Recently, genotype D has become the predominantly circulating lineage globally, with subgenotypes D2 and D3 widely distributed across China. The subgenotype D3 is further subdivided into D3a, D3b and D3c [15].

Recombination is a common feature in the genome of EVs. Due to genomic variation and recombination, 28 recombinant forms (RF) have been found in CV-A6 strains worldwide [18]. Specific genomic regions from different viral lineages may influence pathogenicity. Therefore, monitoring the genetic variations of CV-A6 is essential for identifying novel recombination events and assessing risks related to pathogenicity and transmission.

In this study, the genotype and phylogeny of CV-A6, the main pathogen causing HFMD, were analyzed in Baotou, Inner Mongolia, during 2023–2024.

## 2. Materials and Methods

### 2.1. Sample Collection

We collected 266 throat swabs from patients diagnosed with HFMD at the outpatient departments of hospitals by the Baotou Center for Disease Control and Prevention, Inner Mongolia Autonomous Region, from November 2023 to October 2024. After collection, all samples were stored at −80 °C for subsequent analysis. Among the 266 HFMD cases, 172 were males and 94 were females. The age range of patients was between 1 and 43 years; 98 were aged ≤6 years, and 108 were between 4 and 6 years of age.

### 2.2. EV Identification and Amplification

Viral nucleic acid was extracted from 200 μL of the above samples with the QIAamp MinElute Virus Spin Kit (Qiagen, Hilden, Germany). Enteroviruses were identified using a one-step RT qPCR method. The primers and probes were as follows: forward primer (5′-CCCTGAATGCGGCTAATCC-3′), reverse primer (5′-ATTGTCACCATAAGCAGCCA-3′), and probe (5′-AACCGACTACTTTGGGTGTCCGTGTTTC-3′) (Sangon Biotech, Shanghai, China).

cDNA was synthesized from EV qRT-PCR-positive nucleic acids using a reverse transcription kit (Vazyme, Nanjing, China). The VP1 genes of EV were amplified using semi-nested PCR with the Premix Taq PCR Kit (TAKARA, Tokyo, Japan).

Semi-nested PCR was performed to amplify a partial VP1 gene with the Premix Taq PCR Kit (TAKARA, Japan). Two rounds of primers were used [4]: the first round (forward: 5′-GCIATGYTIGGIACICAYRT-3′, reverse: 5′-CICCIGGIGGIAYRWACAT-3′) and the second round (forward: 5′-CCAGCACTGACAGCAGYNGARAYNGG-3′, reverse: 5′-TACTGGACCACCTGGNGGNAYRWACAT-3′). PCR products were Sanger-sequenced by Tsingke Biotechnology Co., Ltd. (Beijing, China), and sequences were assembled using SeqMan v7.1.0 software. Post-assembly, sequences were aligned via BLAST on the NCBI website (https://blast.ncbi.nlm.nih.gov).

Based on preliminary identification, specific primers were used to amplify the full-length VP1 gene (915 bp) [19]: forward primers 5′-AGAYACCCCCACTGAGGCTAA-3′ and 5′-GAGTGGCGAGATGTCGGTTTAC-3′.

Primers (CV-A6-1~10) were designed for CV-A6 near-complete genome amplification via the primer walking strategy in SnapGene v6.0.2. The 5′/3′ RACE Kit was used to fill in the untranslated regions (Table S1).

### 2.3. Collection of Sequence Data and Evolutionary Analysis

We downloaded 5711 CV-A6 VP1 reference sequences from NCBI (as of April 2025). Sequences were screened with SeqKit v2.10.1 and CD-HIT v4.8.1 (sequence similarity clustering threshold of 0.96 for de-replication) to exclude low-quality, incomplete sequences and indels. A total of 190 reference strains were retained (145 from 25 Chinese provinces) from between 1992 and 2023. In total, 323 strains were obtained, including 133 strains from this study (Table S2). Sequences were aligned with MAFFT v7.520, and a phylogenetic tree was constructed in MEGA v7.0.26 via the maximum likelihood (ML) method with the Kimura 2-parameter model with gamma distribution and invariant sites model (K2 + G + I) (1000 bootstrap replicates). The tree was visualized using FigTree v1.4.4 and iTOL (https://itol.embl.de/, accessed on 16 July 2025).

### 2.4. Amino Acid Similarity and Variation Site Analysis

NT/AA similarity was analyzed with MegAlign v7.1.0 and BioAider v1.727.

### 2.5. Recombination Analysis

Reference sequences of CV-A6 with different RFs were downloaded to construct a phylogenetic tree with the Baotou sequences, and their clustering patterns were analyzed (Table S3). Similarity analysis was performed using Simplot v.3.5.1.

### 2.6. Selection Pressure Analysis

To systematically characterize selection pressures acting on the proteins encoded by the CV-A6 genome, we employed four widely used algorithms available on the Datamonkey platform: Mixed Effects Model of Evolution (MEME), Fixed Effects Likelihood (FEL), Fast Unconstrained Bayesian AppRoximation (FUBAR), and Single Likelihood Ancestor Counting (SLAC). The ratio of non-synonymous substitutions (dN) to synonymous substitutions (dS), designated as ω (ω = dN/dS), was used as the primary metric. Selection pressure was assessed across the entire open reading frame (ORF) of the CV-A6 genome.

### 2.7. Statistical and Bioinformatics Analysis

Data analysis was performed using Excel 2021 and SPSS 27.0 software. The age and sex distribution of all HFMD cases was described. Subsequently, the positivity rates of EV and CV-A6 were analyzed by age, sex, month of detection, and distribution across the nine districts, banners, and counties in Baotou. Categorical variables were analyzed using the chi-square test or Fisher’s exact test, and the Cochran–Armitage test for trends was used to assess age-related trends. *p* < 0.05 was considered statistically significant.

## 3. Results

### 3.1. Characteristic of CV-A6 Cases

Among the 266 HFMD cases, the Cochran–Armitage test for trend showed no statistically significant difference in gender distribution among different age groups (Cochran–Armitage χ^2^ = 0.965, *p* = 0.326). Although the proportion of male cases was relatively high in this study, statistical analysis showed that sex had no significant effect on the risk of EV or CV-A6 infection (*p* > 0.05) (Table 1).

Based on age distribution characteristics, combined with the overall sample and virus detection results, all cases were divided into five age groups: infant group (0–3 years), preschool group (4–6 years), school-age group (7–10 years), adolescent group (11–17 years), and adult group (≥18 years).

**Figure 1:**
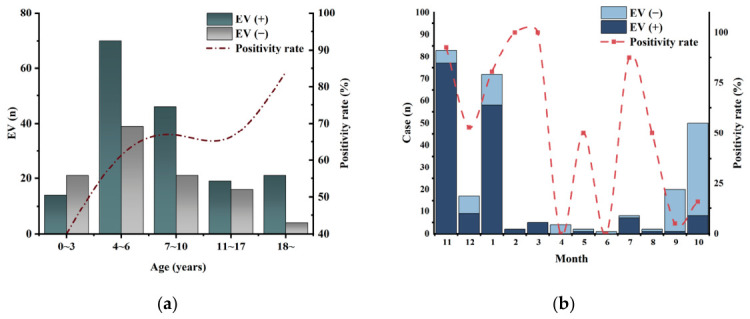
Age group distribution (**a**) and monthly distribution (**b**) of EV-positive cases in Baotou.

The Cochran–Armitage trend test indicated differences in EV positivity rates and CV-A6 positivity rates across age groups, with the preschool group being the high-incidence age group for HFMD, and the difference was statistically significant (*p* < 0.01). The highest number of cases and positivity rate were observed in the 6-year age group, with a positivity rate of 67.57% (25/37), followed by the 4-year age group with a positivity rate of 69.44% (25/36) (Figure 1a). For the area of residence, information was missing for 4 patients, and 4 patients were permanent residents outside Baotou. Due to the presence of theoretical frequencies less than 5 in the spatial distribution analysis, Fisher’s exact test was used for comparison. EV- and CV-A6-positive cases were mainly concentrated in Kundulun District of Baotou (*p* < 0.001). The temporal distribution showed significant differences across seasons (*p* < 0.01), with a clear epidemic pattern characterized by high incidence in autumn and winter. The main peak of EV cases occurred in November, with a secondary peak in January of the following year (Figure 1b). The overall distribution of EV- and CV-A6-positive samples was similar across sex, age, month of detection, and region.

### 3.2. Phylogenetic Analysis of CV-A6 VP1

#### 3.2.1. Phylogenetic Analysis

**Figure 2:**
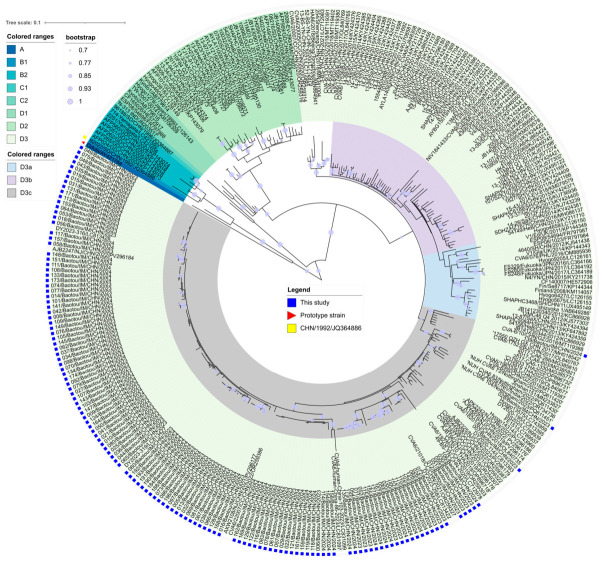
Phylogenetic tree based on CV-A6 VP1 gene sequences. (A phylogenetic tree (ML) was constructed for the VP1 gene region using 323 CV-A6 sequences selected from the sequence database. A total of 1000 bootstrap replicates were performed to assess the robustness of the clades, with bootstrap values ≥ 70% considered reliable for clade assignment).

Phylogenetic analysis of the VP1 gene sequences (Figure 2) showed that all 133 CV-A6 isolates from Baotou (GenBank: PZ060029–PZ060161) clustered within subgenotype D3c, the currently predominant lineage globally. The reference sequences representing other genotypes (A, B, C, D1, D2, D3a, D3b) formed distinct clades as expected, but no Baotou isolates fell into these clades.

#### 3.2.2. Similarity Analysis

The VP1 gene of CV-A6 comprises 915 NTs, encoding 305 amino acids. VP1 sequence identity was analyzed among 133 CV-A6 isolates from this study and eight reference strains with different genotypes/subgenotypes, years, and geographic regions.

**Figure 3:**
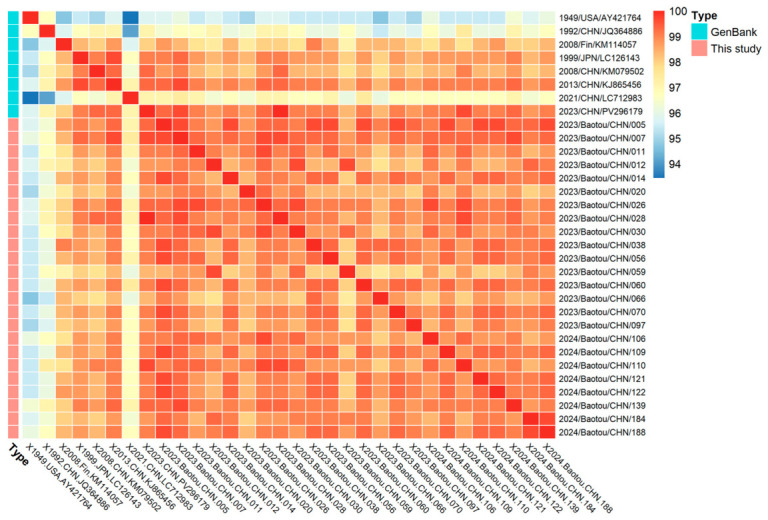
Heatmap of amino acid sequence similarity for CV-A6 VP1 (Amino acid identity heatmap was generated by aligning the VP1 gene-derived amino acid sequences of CV-A6 strains from Baotou with those of CV-A6 reference strains from different years. The color intensity in the heatmap directly indicates the level of amino acid identity. Darker colors represent higher identity, while lighter colors represent lower identity).

The Baotou isolates exhibited high intra-strain sequence conservation, with NT identities ranging from 92.35% to 100% and amino acid (AA) identities from 97.38% to 100%. Comparison with reference strains revealed that the Baotou isolates shared 82.08–83.5% NT and 94.75–96.39% AA identity with the prototype strain Gdula (1949/USA/AY421764); 82.3–84.15% NT and 95.41–97.38% AA identity with China’s first reported epidemic strain (1992/SD/CHN/JQ364886); and moderately higher identity (91.04–92.79% NT, 97.70–98.03% AA) with the earliest D3 subgenotype strain (2008/Finland/KM114057) (Figure 3). Notably, Baotou isolates exhibited the highest NT identity (99.78%) with recent D3c subgenotype strains from Sichuan, China (2023/LC813426), confirming their close genetic correlation. Collectively, these results indicate that CV-A6 strains from Baotou have undergone genetic divergence from early reference strains (prototype strain, China’s first epidemic strain, and other D3 subgenotype strains), while maintaining high homology among local isolates and clustering with contemporary D3c subgenotype strains.

#### 3.2.3. Amino Acid Variation Site Analysis of CV-A6

Amino acid variation analysis identified eleven fixed substitutions (present in 100% of local isolates) between 133 CV-A6 isolates from Baotou and the prototype strain Gdula: S5T, I8V, N10S, T14A, S32T, Q98L, I174V, S194T, F261L, S279T, and F305S. Notably, two additional fixed substitutions—including the clade-defining T283A mutation—were detected in Baotou strains relative to China’s first D3 subgenotype strain (KM079502, early D3 lineage), consistent with their classification into the contemporary D3c clade.

#### 3.2.4. Phylogenetic Analysis Based on CV-A6 Full-Genome Sequences

Phylogenetic trees of the ORF, P1, P2, and P3 regions of CV-A6 were constructed using sequences of CV-A6 strains exceeding 6603 bp retrieved from the sequence database together with those of the Baotou CV-A6 strains, of which only a single representative sequence was retained when NT identity reached 100% among sequences. Phylogenetic analysis revealed that the NT that the identity of ORF, P1, P2 and P3 regions ranged from 79.34% to 99.98%, 81.72% to 99.96%, 78.37% to 100%, and 76.32% to 100%, respectively. These results suggest that the P2/P3 regions, which encode non-capsid proteins, exhibit higher genetic diversity, with frequent recombination events observed in the non-capsid regions of CV-A6.

**Figure 4:**
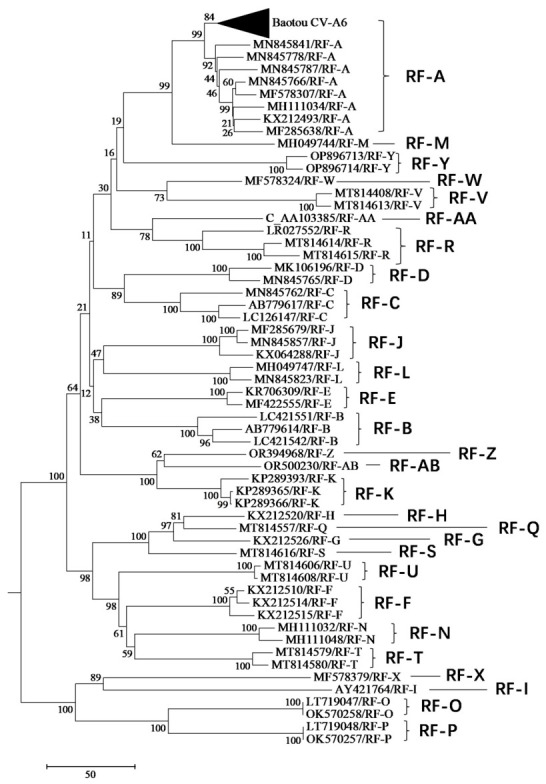
The RF of CV-A6 isolates from Baotou (A phylogenetic tree (ML) was constructed using the 3D gene region sequences of 29 CV-A6 strains from Baotou and representative strains of different RFs, aiming to analyze the recombinant lineage affiliation of Baotou strains).

Previous studies have classified CV-A6 recombination types into 28 categories based on the 3D region [10,20]. To clarify the recombination profile of Baotou strains, phylogenetic trees were constructed using 3D gene sequences of 29 CV-A6 full-genome isolates obtained in this study (GenBank: PZ094945-PZ094973), alongside 3D gene sequences of reference strains representing different recombination clades (Table S2). Phylogenetic trees were constructed separately for the non-capsid regions P2 and P3, as well as the recombination-prone 3C-3D region, and the Baotou isolates consistently clustered within the RF-A branch (Figure S1). Similarity analysis was performed using Simplot v.3.5.1 (referencing Gdula, positions 746–7348 bp) with a sliding window of 200 NT and a step size of 20 NT, and the Baotou isolates consistently showed high similarity to RF-A (Figure S2), indicating that all CV-A6 strains circulating in Baotou belong to the RF-A lineage, with no emergence of novel recombinant forms. Results showed that all Baotou CV-A6 sequences clustered within the RF-A lineage (Figure 4).

#### 3.2.5. Result of Selection Pressure Analysis

In this study, the selection pressures acting on the amino acid sequences encoded by the complete ORF of CV-A6 were systematically analyzed using four algorithms available on the Datamonkey platform: MEME, FEL, FUBAR, and SLAC. The results showed that amino acid position 807 in the ORF (corresponding to residue 242 of the VP1 protein) was identified as a positively selected site by three algorithms simultaneously—MEME, FEL, and FUBAR, suggesting sustained diversifying selection at this locus during viral evolution. Conversely, multiple negatively selected sites were found to be under strong purifying selection by FEL, FUBAR, and SLAC (Table S4). Collectively, these results reveal that, although a few sites show evidence of adaptive evolution, the CV-A6 ORF is predominantly governed by purifying selection, which maintains the structural and functional stability of the viral proteins.

## 4. Discussion

HFMD remains a major public health concern with global implications. Since its inclusion in China’s statutory infectious disease surveillance system in 2008, the etiological spectrum of HFMD has continuously evolved. CV-A6 has emerged as a predominant pathogen causing atypical HFMD in recent years [21,22,23], with pathogenic mechanisms distinct from traditional agents such as EV-A71 and CV-A16—for example, it exhibits unique characteristics in inducing apoptosis [24]. Initially spreading across European countries (e.g., France, Spain) between 2009 and 2011, CV-A6 has subsequently caused outbreaks in multiple regions of mainland China, establishing its status as a major etiological agent of HFMD.

This study analyzed the prevalence of CV-A6 in Baotou from November 2023 to October 2024, representing the first report of CV-A6-driven HFMD outbreaks in Inner Mongolia in recent years. Epidemiological analysis confirmed CV-A6 as the predominant HFMD pathogen in Baotou during the study period. The male-to-female ratio of cases was 1.83:1, higher than reported in previous studies [20,21,22,23]; however, statistical analysis showed gender was not a significant risk factor for CV-A6 infection (*p* > 0.05). Regarding age distribution, the infection peak occurred in children aged 4–6 years, potentially attributable to the persistence of maternal antibodies in children aged 1–3 years [22]. Notably, in contrast to the traditional pattern of HFMD predominantly affecting children under 5 years old [6,23,25], a considerable number of older children and adults were infected in this outbreak—suggesting enhanced infectivity of the current prevalent strain to older age groups.

In terms of temporal distribution, the epidemic in this study peaked in autumn and winter, deviating from the typical spring–summer seasonality of HFMD in temperate regions [22,24]. In addition to viral factors, this change may be partially attributed to the implementation of non-drug interventions (NPIs) during the COVID-19 pandemic, which interfered with the transmission of enteroviruses. After the withdrawal of NPIs at the end of 2022, enteroviruses, especially CV-A6, showed compensatory recovery, a phenomenon also documented in other countries [26].

CV-A6 has replaced EV-A71 as the predominant serotype in many regions [19,26,27], and our findings align with this broader epidemiological shift, indicating that Baotou is not an isolated case but part of a nationwide trend. Nationwide surveillance data from the same period further support this notion: in Ningxia, the autumn–winter peak in 2023 exceeded the spring–summer peak for the first time on record [28]; in Beijing, the peak date was delayed by 54 days compared with pre-2016 seasons [29]; and in Zhejiang Province, the 2023 summer peak was higher than in previous years and delayed by approximately three weeks [30]. A national-level analysis confirmed that the 2023 spring–summer HFMD peak was significantly elevated and occurred later than usual [31]. These consistent patterns across multiple provinces suggest that the change in enterovirus circulation was a nationwide phenomenon, likely driven by the accumulation of susceptible individuals during the NPI period and the predominance of CV-A6. The use of EV-A71 vaccines and the impact of the COVID-19 pandemic may have jointly promoted the changes in the etiological spectrum of HFMD [31].

Spatially, cases were markedly clustered in Kundulun District, a pattern likely driven by local factors such as population density and social activity patterns [24]. As an inland city in northern China, Baotou has a temperate continental monsoon climate—characterized by aridity, low precipitation, and large diurnal temperature differences—which contrasts with the humid, hot regions traditionally associated with high HFMD incidence [23]. This unique spatiotemporal distribution suggests that in this CV-A6-dominated epidemic, the virus’s enhanced transmissibility and host infectivity (e.g., increased susceptibility of school-age children and adults) may have partially overridden climatic constraints, leading to changes in seasonal epidemic pattern. These findings highlight that HFMD epidemiological characteristics are jointly shaped by the dominant pathogen strain, viral evolution, macro-geographical factors, and climate, underscoring the need to integrate regional specificity and viral variation into prevention and control strategies.

To further characterize the genetic features of the circulating strains, a phylogenetic tree was constructed based on the complete VP1 gene sequences, given that the VP1 coding region contains key neutralizing epitopes and enables accurate serotype identification of enteroviruses. The results showed that 133 Baotou isolates clustered closely with strains from other regions of China within the subgenotype D3c clade, consistent with the domestic epidemiological trend over the past five years. These strains exhibited substantial genetic distance from reference strains (e.g., the prototype strain Gdula), indicating that the CV-A6 strains prevalent in Baotou share a single origin and exhibit low genetic variation. In summary, the subgenotype D3c exhibits strong adaptability and transmission capacity.

As with many EVs, such as CV-A16 and EV-A71, CV-A6 typically binds to receptors on the cell surface through highly exposed surface loops of capsid proteins. A study summarized the BC, DE, EF, and HI surface loops as potential receptor-binding sites in the VP1 of CV-A6 [28]. Amino acid site variations may enhance the receptor-binding capacity of the virus. The variations at sites such as V174I, T283A, A5T, V30A, S137N, and I242V may have contributed to CV-A6 outbreaks in Guangxi, China [32], suggesting that continuous monitoring of amino acid variations is crucial for epidemic prevention and control. The differences in the evolutionary patterns of strains observed in this study compared with those in other regions indicate that prevalent CV-A6 strains and their evolutionary characteristics may exhibit geographical differentiation.

Focusing on key amino acid substitutions, compared with the prototype strain, CV-A6 in Baotou has undergone multiple variations, which may have important biological significance. The I174V variation is located in the EF loop; previous studies have con-firmed that variations at this site may interfere with viral binding or adsorption to host cells, and this substitution has been detected in prevalent strains in multiple regions [28,33]. The mutation at VP1-137 is located within the DE loop of the putative receptor-binding site and is speculated to potentially affect the pathogenicity of the strain [34]. This mutation is characterized by an S137N substitution, and the structure of S137N is observed to adopt a bent conformation, which may influence the virulence of the CV-A6 virus. Meanwhile, the amino acid entropy at the VP1-137 site is calculated to be greater than 0.6, indicating that it is a highly variable site within the VP1 region of CV-A6 and is therefore considered to possess strong evolutionary plasticity and genetic diversity. Additionally, the V242I variation in the HI loop, recently identified in the RF-AA strain from Beijing, was detected in 5 cases in this study. This variation is also located within the putative receptor-binding site region, suggesting potential involvement in the regulation of viral pathogenicity. VP1-242 (ORF-807) was also the only positively selected site identified in this study, and previous studies have suggested that this site may enhance the adaptation of CV-A6 to the host [20,34]. This indicates that the evolution of CV-A6 has been mainly driven by purifying selection to maintain the structural stability of the protein, while the VP1-242 site may represent a rare adaptive evolutionary hotspot. This site is located in the putative receptor-binding region (HI loop), and its positive selection signal suggests a possible association with host adaptation or immune evasion. VP1-283 represents one of the globally highly variable sites in VP1 and was found to be a 100% variable site when comparing Baotou strains with KM079502 (the first D3 prevalent strain in China). Previous studies have demonstrated that a substitution from hydrophilic threonine (Thr) to hydrophobic alanine (Ala) occurs at VP1-283, which may determine the clustering of genotypes D3a–D3c. When occupied by alanine, this residue forms a hydrogen bond with VP3-66 [18]. Collectively, these substitutions occur at antigenic epitopes or receptor-binding loops (EF, DE, and HI loops) of known functional importance, reinforcing their relevance for viral evolution, host adaptation, and epidemiological surveillance.

In addition to point mutations, recombination analysis confirmed that all Baotou CV-A6 isolates belonged to the known RF-A lineage, with no novel recombinant form detected. This finding, while not unexpected given the widespread predominance of RF-A in China, provides valuable baseline data for future surveillance of recombinational emergence. The continued circulation of RF-A in Baotou aligns with national trends, indicating genetic stability of the circulating strains during the study period. However, the promotion of EV-A71 vaccines and the impact of the COVID-19 pandemic may have jointly driven changes in the etiological spectrum of HFMD [35]. Therefore, continuous monitoring of the epidemiological dynamics and pathogenic variations in CV-A6 is essential for understanding the epidemiological characteristics of HFMD and formulating evidence-based prevention and control strategies.

This study has several limitations. First, the sample size was relatively small and the representation of other enterovirus genotypes was limited, precluding comprehensive comparison between CV-A6 and other genotypes. Second, as this is the first report of HFMD in Baotou, no previous local surveillance data on pathogen spectra or CV-A6 molecular epidemiology are available, precluding assessment of long-term evolutionary trends. Third, detailed clinical information (symptoms, rash morphology, disease progression) was not systematically collected, which prevented us from correlating specific genotypes or variants with clinical manifestations. Despite these limitations, the observed epidemic trend was consistent with reports from other regions in China [18,19,29,32,36]. Continuous monitoring of CV-A6 dynamics remains essential for evidence-based prevention and control.

## 5. Conclusions

During 2023–2024, CV-A6 was the predominant pathogen of HFMD in Baotou, ac-counting for 85.88% of EV-positive cases. The epidemic peaked in autumn and winter, with children aged 4–6 years being the most affected group. All 133 CV-A6 isolates belonged to subgenotype D3c and the RF-A recombinant lineage, consistent with the strains circulating nationally. Our results provided baseline data for future monitoring of recombinational shifts. This study fills a critical data gap for Baotou, Inner Mongolia, offering the first evidence-based reference for local HFMD prevention and control.

## Figures and Tables

**Table 1:** Overall distribution of EV-positive and CV-A6-positive samples by gender, age, month and region in Baotou.

	EV Positive	EV Positive%	*χ*^2^/Fisher/Cochran–Armitage *χ*^2^	*p*	CV-A6 Positive	A6 Positive%	*χ*^2^/Fisher/Cochran– Armitage *χ*^2^	*p*	Total
Gender									
Male	108	62.79%	0.26	0.60	97	56.40%	0.45	0.50	172
Female	62	65.96%			49	52.13%			94
Age									
0~3	14	40.00%	7.85	**	13	37.14%	8.69	**	35
4~6	70	64.81%			55	50.93%			108
7~10	46	68.66%			41	61.19%			67
11~17	19	61.29%			19	61.29%			31
≥18	21	84.00%			18	72.00%			25
Season									
Spring	6	54.55%	11.48	**	1	9.09%	21.05	***	11
Summer	9	81.82%			7	63.64%			11
Autumn	86	56.21%			74	48.37%			153
Winter	69	75.82%			64	70.33%			91
Region									
Kundulun District	110	83.33%	Fisher	***	95	71.97%	Fisher	***	132
Qingshan District	12	40.00%			10	33.33%			30
Donghe District	23	52.27%			19	43.18%			44
Jiuyuan District	8	25.81%			8	25.81%			31
Guyang County	8	57.14%			7	50.00%			14
Shiguai District	1	25.00%			1	25.00%			4
Tumote Youqi	1	100.00%			1	100.00%			1
Damaoqi	1	100.00%			1	100.00%			1
Baiyun Kuangqu	0	0.00%			0	0.00%			1
Other Region	4	100.00%			3	75.00%			4
Unknown	2	50.00%			1	25.00%			4
Total	170	63.91%			146	54.89%			266

** *p* < 0.01, *** *p* < 0.001.

## Data Availability

The original contributions presented in this study are included in the article/Supplementary Materials. Further inquiries can be directed to the corresponding author.

## Supplementary Materials

The following supporting information can be downloaded at: https://www.mdpi.com/article/10.3390/v18060680/s1, Table S1: CV-A6 whole genome amplification primers; Table S2: Information on 323 CV-A6 for phylogenetic analysis; Table S3: Reference sequences of different RFs; Table S4: Selection pressure analysis of the CV-A6 ORF using four algorithms; Figure S1: Phylogenetic analysis using the maximum-likelihood method with 1000 bootstrap replicates was performed on the non-capsid protein regions of Baotou isolates (removing duplicates of identical sequences): (A) P2 region; (B) P3 region; (C) 3C-3D region; (D) P2-P3 region; Figure S2: Similarity analysis of Baotou isolates was performed using a sliding window of 200 nt with a step size of 20 nt.

## Abbreviations

The following abbreviations are used in this manuscript:

AA	Amino acid
CDR	Complementarity-determining region
CV-A6	Coxsackievirus A6
HFMD	Hand, foot, and mouth disease
EV	Enterovirus
ML	Maximum likelihood
NT	Nucleotide
ORF	Open reading frame
RF	Recombinant forms
Thr	Threonine
