# Supplementary material for: Epidemiological Characteristics of Coxsackievirus A6 in Baotou, Inner Mongolia, China, 2023–2024
Source: Viruses. 2026 Jun 18;18(6):680. doi: 10.3390/v18060680 (PMC13307889; doi:10.3390/v18060680)
Supplement: Supplementary file 1 [file viruses-18-00680-s001.zip › viruses-Supplementary_Material.pdf]

Supplementary Material

1 Supplementary Tables

Table S1.CV-A6 whole genome amplification primers

Primer	Sequence(5'-3')	Orientation
CVA6VP1-F1	CGGGATCTCACCATTTGG	Forward
CVA6VP1-R	CRAGATGGTCGGTTTACCACTCT	Reverse
5′R	CGCACGCCTCAACAGAAGGGGATTG	Reverse
CVA6-1F	CTACGGAATCTTTGTGCG	Forward
CVA6-1R	TACAATGTTGGCTGCCTC	Reverse
CVA6-2F	AGCGTCAGCTAGTAGACA	Forward
CVA6-2R	TGCCAGTGGTGTTATTGAC	Reverse
CVA6-3F	TCACACTAACTATTGCCCC	Forward
CVA6-3R	ACCCCGTTTCGATTCATC	Reverse
CVA6-4F	TGACGAGATCCAGCAGAC	Forward
CVA6-4R	TTACCACTCTGAAGTTGCC	Reverse
CVA6-5F	CACGTTCGGGTATACATGA	Forward
CVA6-5R	CACTCAAATCCCTTGGCA	Reverse
CVA6-6F	GGGGCTGTTGAAAAGATC	Forward
CVA6-6R	CCACACACTAGTGGGCTA	Reverse
CVA6-7F	TATCGCATCCACTAATGCC	Forward
CVA6-7R	CACTGAGATTCAGGAAACC	Reverse
CVA6-8F	CTCGTGAACATCCTGGAT	Forward
CVA6-8R	GAACCAGTCACAGTCCCA	Reverse
CVA6-9F	CCACCAGGGATGTGAGTA	Forward
CVA6-9R	GTGGGGTTGAGGTGTATTC	Reverse
CVA6-10F	TCACGAATCCATTCGGTG	Forward
CVA6-10R	GCTATTCTGGTTATAACA	Reverse
3′F	ACCTCAGAATGGCCTTCGGGCATCTC	Forward

Table S2 Information on 323 CVA6 for phylogenetic analysis

GenBank accession No.	Origion	Countries	Isolated Year	GenBank accession No.	Origion	Countries	Isolated Year
AY421764	GenBank	USA	1949	OR259314	GenBank	CHN	2020
JQ364886	GenBank	CHN	1992	LC813393	GenBank	CHN	2020
JQ364887	GenBank	CHN	1996	LC813395	GenBank	CHN	2020
LC126143	GenBank	JPN	1999	OR259327	GenBank	CHN	2021
AB779614	GenBank	JPN	1999	OR259322	GenBank	CHN	2021
LC421656	GenBank	JPN	2002	LC743960	GenBank	JPN	2021
LC421592	GenBank	JPN	2002	LC712983	GenBank	CHN	2021
AB779616	GenBank	JPN	2003	LC712985	GenBank	CHN	2021
KP143073	GenBank	CHN	2004	PV296177	GenBank	CHN	2021
KP143074	GenBank	CHN	2004	PQ611430	GenBank	UK	2022
KP143075	GenBank	CHN	2005	PQ611420	GenBank	UK	2022
KP143076	GenBank	CHN	2005	OR734736	GenBank	IND	2022
KP143077	GenBank	CHN	2006	OP896731	GenBank	THA	2022
KP143078	GenBank	CHN	2007	OP896732	GenBank	THA	2022
KP143079	GenBank	CHN	2007	OR259387	GenBank	CHN	2022
KP143081	GenBank	CHN	2007	OR259386	GenBank	CHN	2022
LC126147	GenBank	JPN	2007	PV296184	GenBank	CHN	2022
KR706309	GenBank	CHN	2007	PV296186	GenBank	CHN	2022
KP144344	GenBank	Fin	2008	PV296176	GenBank	CHN	2022
KM114057	GenBank	Fin	2008	PV296179	GenBank	CHN	2023
KM079502	GenBank	CHN	2008	PQ611451	GenBank	UK	2023
KC866900	GenBank	CHN	2008	LC831099	GenBank	CHN	2023
KC866901	GenBank	CHN	2008	LC831097	GenBank	CHN	2023
JN203517	GenBank	IND	2008	LC813427	GenBank	CHN	2023
JQ364888	GenBank	CHN	2008	LC813426	GenBank	CHN	2023
FR797988	GenBank	ESP	2008	LC813422	GenBank	CHN	2023

GenBank accession No.	Origion	Countries	Isolated Year	GenBank accession No.	Origion	Countries	Isolated Year
FR797984	GenBank	ESP	2008	LC789939	GenBank	JPN	2023
FR797987	GenBank	ESP	2008	LC789937	GenBank	JPN	2023
LC126149	GenBank	JPN	2009	PZ060029	This study	CHN	2023
LC126150	GenBank	JPN	2009	PZ060030	This study	CHN	2023
KM079512	GenBank	CHN	2009	PZ060031	This study	CHN	2023
KC866921	GenBank	CHN	2009	PZ060032	This study	CHN	2023
KC866916	GenBank	CHN	2009	PZ060033	This study	CHN	2023
KM079511	GenBank	CHN	2009	PZ060034	This study	CHN	2023
JQ946050	GenBank	CHN	2009	PZ060035	This study	CHN	2023
HE572917	GenBank	FRA	2010	PZ060036	This study	CHN	2023
HE572928	GenBank	FRA	2010	PZ060037	This study	CHN	2023
HE572906	GenBank	FRA	2010	PZ060038	This study	CHN	2023
KJ865427	GenBank	CHN	2010	PZ060039	This study	CHN	2023
KJ865424	GenBank	CHN	2010	PZ060040	This study	CHN	2023
LC126153	GenBank	JPN	2010	PZ060041	This study	CHN	2023
KJ577273	GenBank	CHN	2010	PZ060042	This study	CHN	2023
KJ577274	GenBank	CHN	2010	PZ060043	This study	CHN	2023
JQ364889	GenBank	CHN	2010	PZ060044	This study	CHN	2023
JX495122	GenBank	CHN	2010	PZ060045	This study	CHN	2023
AB649286	GenBank	JPN	2011	PZ060046	This study	CHN	2023
KJ743209	GenBank	CHN	2011	PZ060047	This study	CHN	2023
KJ743212	GenBank	CHN	2011	PZ060048	This study	CHN	2023
KY211690	GenBank	CHN	2011	PZ060049	This study	CHN	2023
KM079578	GenBank	CHN	2011	PZ060050	This study	CHN	2023
KY211707	GenBank	CHN	2011	PZ060051	This study	CHN	2023
KU708571	GenBank	CHN	2011	PZ060052	This study	CHN	2023
LC126155	GenBank	JPN	2011	PZ060053	This study	CHN	2023

GenBank accession No.	Origion	Countries	Isolated Year	GenBank accession No.	Origion	Countries	Isolated Year
KJ609186	GenBank	CHN	2011	PZ060054	This study	CHN	2023
KJ577298	GenBank	CHN	2011	PZ060055	This study	CHN	2023
JX495130	GenBank	CHN	2011	PZ060056	This study	CHN	2023
KJ541166	GenBank	CHN	2011	PZ060057	This study	CHN	2023
JX495149	GenBank	CHN	2011	PZ060058	This study	CHN	2023
KY211724	GenBank	CHN	2011	PZ060059	This study	CHN	2023
KP144349	GenBank	UK	2011	PZ060060	This study	CHN	2023
MH049745	GenBank	CHN	2012	PZ060061	This study	CHN	2023
KJ743239	GenBank	CHN	2012	PZ060062	This study	CHN	2023
KM079579	GenBank	CHN	2012	PZ060063	This study	CHN	2023
KC866928	GenBank	CHN	2012	PZ060064	This study	CHN	2023
KC866929	GenBank	CHN	2012	PZ060065	This study	CHN	2023
KY211711	GenBank	CHN	2012	PZ060066	This study	CHN	2023
KU708572	GenBank	CHN	2012	PZ060067	This study	CHN	2023
KJ577302	GenBank	CHN	2012	PZ060068	This study	CHN	2023
KJ577303	GenBank	CHN	2012	PZ060069	This study	CHN	2023
KJ541169	GenBank	CHN	2012	PZ060070	This study	CHN	2023
KJ541438	GenBank	CHN	2012	PZ060071	This study	CHN	2023
KP144350	GenBank	UK	2012	PZ060072	This study	CHN	2023
KP144341	GenBank	UK	2012	PZ060073	This study	CHN	2023
KY424425	GenBank	CHN	2012	PZ060074	This study	CHN	2023
KJ743243	GenBank	CHN	2013	PZ060075	This study	CHN	2023
KY211689	GenBank	CHN	2013	PZ060076	This study	CHN	2023
KJ865456	GenBank	CHN	2013	PZ060077	This study	CHN	2023
KF647892	GenBank	CHN	2013	PZ060078	This study	CHN	2023
KY424405	GenBank	CHN	2013	PZ060079	This study	CHN	2023
KY211710	GenBank	CHN	2013	PZ060080	This study	CHN	2023

GenBank accession No.	Origion	Countries	Isolated Year	GenBank accession No.	Origion	Countries	Isolated Year
KY424359	GenBank	CHN	2013	PZ060081	This study	CHN	2023
KY424393	GenBank	CHN	2013	PZ060082	This study	CHN	2023
KU366286	GenBank	IND	2013	PZ060083	This study	CHN	2023
LC126161	GenBank	JPN	2013	PZ060084	This study	CHN	2023
KJ577316	GenBank	CHN	2013	PZ060085	This study	CHN	2023
KY424418	GenBank	CHN	2013	PZ060086	This study	CHN	2023
KY424390	GenBank	CHN	2013	PZ060087	This study	CHN	2023
KY424420	GenBank	CHN	2013	PZ060088	This study	CHN	2023
KY424421	GenBank	CHN	2013	PZ060089	This study	CHN	2023
KY424408	GenBank	CHN	2013	PZ060090	This study	CHN	2023
KY424388	GenBank	CHN	2013	PZ060091	This study	CHN	2023
KY424394	GenBank	CHN	2013	PZ060092	This study	CHN	2023
KY211691	GenBank	CHN	2013	PZ060093	This study	CHN	2023
KJ541369	GenBank	CHN	2013	PZ060094	This study	CHN	2023
KY211727	GenBank	CHN	2013	PZ060095	This study	CHN	2023
KY424424	GenBank	CHN	2013	PZ060096	This study	CHN	2023
KJ848296	GenBank	CHN	2013	PZ060097	This study	CHN	2023
KJ848308	GenBank	CHN	2013	PZ060098	This study	CHN	2023
KP144339	GenBank	UK	2013	PZ060099	This study	CHN	2023
KY424406	GenBank	CHN	2013	PZ060100	This study	CHN	2024
KY424404	GenBank	CHN	2013	PZ060101	This study	CHN	2024
KY424358	GenBank	CHN	2013	PZ060102	This study	CHN	2024
KY211737	GenBank	CHN	2013	PZ060103	This study	CHN	2024
KY424409	GenBank	CHN	2013	PZ060104	This study	CHN	2024
KY211692	GenBank	CHN	2014	PZ060105	This study	CHN	2024
KU708581	GenBank	CHN	2014	PZ060106	This study	CHN	2024
KY424386	GenBank	CHN	2014	PZ060107	This study	CHN	2024

GenBank accession No.	Origion	Countries	Isolated Year	GenBank accession No.	Origion	Countries	Isolated Year
KY424385	GenBank	CHN	2014	PZ060108	This study	CHN	2024
KY424382	GenBank	CHN	2014	PZ060109	This study	CHN	2024
KY424381	GenBank	CHN	2014	PZ060110	This study	CHN	2024
KU212275	GenBank	CHN	2014	PZ060111	This study	CHN	2024
KY424376	GenBank	CHN	2014	PZ060112	This study	CHN	2024
KP144348	GenBank	UK	2014	PZ060113	This study	CHN	2024
KP144343	GenBank	UK	2014	PZ060114	This study	CHN	2024
KY424374	GenBank	CHN	2014	PZ060115	This study	CHN	2024
KY424377	GenBank	CHN	2014	PZ060116	This study	CHN	2024
KY211729	GenBank	CHN	2014	PZ060117	This study	CHN	2024
KY424372	GenBank	CHN	2015	PZ060118	This study	CHN	2024
KY211704	GenBank	CHN	2015	PZ060119	This study	CHN	2024
KY211698	GenBank	CHN	2015	PZ060120	This study	CHN	2024
KU708570	GenBank	CHN	2015	PZ060121	This study	CHN	2024
KY424369	GenBank	CHN	2015	PZ060122	This study	CHN	2024
KY424371	GenBank	CHN	2015	PZ060123	This study	CHN	2024
KY424360	GenBank	CHN	2015	PZ060124	This study	CHN	2024
KU736936	GenBank	CHN	2015	PZ060125	This study	CHN	2024
KY424370	GenBank	CHN	2015	PZ060126	This study	CHN	2024
KY424365	GenBank	CHN	2015	PZ060127	This study	CHN	2024
KY424357	GenBank	CHN	2015	PZ060128	This study	CHN	2024
KY211738	GenBank	CHN	2015	PZ060129	This study	CHN	2024
MH086137	GenBank	CHN	2016	PZ060130	This study	CHN	2024
OM885936	GenBank	PHL	2016	PZ060131	This study	CHN	2024
LC364166	GenBank	JPN	2016	PZ060132	This study	CHN	2024
MG385821	GenBank	CHN	2017	PZ060133	This study	CHN	2024
MG385773	GenBank	CHN	2017	PZ060134	This study	CHN	2024

GenBank accession No.	Origion	Countries	Isolated Year	GenBank accession No.	Origion	Countries	Isolated Year
MG385810	GenBank	CHN	2017	PZ060135	This study	CHN	2024
MH018542	GenBank	CHN	2017	PZ060136	This study	CHN	2024
OL840767	GenBank	CHN	2017	PZ060137	This study	CHN	2024
OL840765	GenBank	CHN	2017	PZ060138	This study	CHN	2024
OL840769	GenBank	CHN	2017	PZ060139	This study	CHN	2024
LC364189	GenBank	JPN	2017	PZ060140	This study	CHN	2024
LC364192	GenBank	JPN	2017	PZ060141	This study	CHN	2024
MT577710	GenBank	IND	2018	PZ060142	This study	CHN	2024
MN864941	GenBank	CHN	2018	PZ060143	This study	CHN	2024
OL840735	GenBank	CHN	2018	PZ060144	This study	CHN	2024
MT119391	GenBank	CHN	2018	PZ060145	This study	CHN	2024
MT119402	GenBank	CHN	2018	PZ060146	This study	CHN	2024
MT119380	GenBank	CHN	2018	PZ060147	This study	CHN	2024
MT119388	GenBank	CHN	2018	PZ060148	This study	CHN	2024
MT119387	GenBank	CHN	2018	PZ060149	This study	CHN	2024
MN233827	GenBank	CHN	2018	PZ060150	This study	CHN	2024
PV296180	GenBank	CHN	2018	PZ060151	This study	CHN	2024
OL840677	GenBank	CHN	2019	PZ060152	This study	CHN	2024
OR259307	GenBank	CHN	2019	PZ060153	This study	CHN	2024
OR259303	GenBank	CHN	2019	PZ060154	This study	CHN	2024
OP896718	GenBank	THA	2019	PZ060155	This study	CHN	2024
LC743959	GenBank	JPN	2019	PZ060156	This study	CHN	2024
MN341004	GenBank	CHN	2019	PZ060157	This study	CHN	2024
PV296174	GenBank	CHN	2019	PZ060158	This study	CHN	2024
PV296178	GenBank	CHN	2019	PZ060159	This study	CHN	2024
LC712979	GenBank	CHN	2020	PZ060160	This study	CHN	2024
LC712978	GenBank	CHN	2020	PZ060161	This study	CHN	2024

GenBank accession No.	Origion	Countries	Isolated Year	GenBank accession No.	Origion	Countries	Isolated Year
OR259316	GenBank	CHN	2020				

Table S3 Reference sequences of different RFs

RFs	ID	Origion	Countries	Year
RF-A	MN845841	GenBank	CHN	2017
	MN845778	GenBank	CHN	2015
	MN845787	GenBank	CHN	2016
	KX212493	GenBank	THA	2014
	MH111034	GenBank	AUS	2016
	MF285638	GenBank	CHN	2014
	MF578307	GenBank	VNM	2011
	MN845766	GenBank	CHN	2014
RF-B	AB779614	GenBank	JPN	1999
	LC421542	GenBank	JPN	2001
	LC421551	GenBank	JPN	2005
RF-C	LC126147	GenBank	JPN	2007
	AB779617	GenBank	JPN	2009
	MN845762	GenBank	CHN	2011
RF-D	MN845765	GenBank	CHN	2012
	MK106196	GenBank	CHN	2013
RF-E	KR706309	GenBank	CHN	2007
	MF422555	GenBank	CHN	2008
RF-F	KX212514	GenBank	THA	2012
	KX212515	GenBank	THA	2014
	KX212510	GenBank	DNK	2014
	KP144352	GenBank	UK	2013

RFs	ID	Origion	Countries	Year
RF-G	KX212526	GenBank	DNK	2014
RF-H	KX212520	GenBank	ESP	2013
	KP144347	GenBank	UK	2014
RF-I	AY421764	GenBank	USA	1949
RF-J	MF285679	GenBank	CHN	2016
	MN845857	GenBank	CHN	2017
	KX064288	GenBank	CHN	2015
RF-K	KP289365	GenBank	CHN	2013
	KP289393	GenBank	CHN	2013
	KP289366	GenBank	CHN	2013
	KM279379	GenBank	CHN	2013
	KT779410	GenBank	CHN	2013
RF-L	MH049747	GenBank	CHN	2015
	MN845823	GenBank	CHN	2017
RF-M	MH049744	GenBank	CHN	2015
RF-N	MH111032	GenBank	AUS	2016
	MH111048	GenBank	AUS	2017
RF-O	OK570258	GenBank	MAD	2011
	LT719047	GenBank	MAD	2011
RF-P	LT719048	GenBank	MAD	2011
	OK570257	GenBank	MAD	2011
RF-Q	MT814557	GenBank	FRA	2010
RF-R	MT814614	GenBank	FRA	2012
	MT814615	GenBank	FRA	2018
	LR027552	GenBank	TKM	2011
RF-S	MT814616	GenBank	FRA	2018
RF-T	MT814580	GenBank	FRA	2010
	MT814579	GenBank	FRA	2010
	MT814608	GenBank	FRA	2011

RFs	ID	Origion	Countries	Year
RF-U	MT814606	GenBank	FRA	2011
RF-V	MT814408	GenBank	FRA	2014
	MT814613	GenBank	FRA	2014
RF-W	MF578324	GenBank	VNM	2012
RF-X	MF578379	GenBank	VNM	2012
RF-Y	OP896714	GenBank	THA	2019
	OP896713	GenBank	THA	2020
RF-Z	OR394968	GenBank	CHN	2023
RF-AB	OR500230	GenBank	CHN	2023
RF-AA	C_AA103385.1	GenBase	CHN	2023

Table S4 Selection pressure analysis of the CV-A6 ORF using four algorithms

Method	Positively selected sites			Negatively selected sites
	AA site (ORF)	P	Posterior prob.	
MEME	193, 260, 424, 517, 668, 777, 807, 896, 941, 990, 1510, 1546, 1785, 1807, 1999, 2001	0.2	-	-
FEL	8, 807, 896	0.1	-	1405
FUBAR	807	-	0.9	1939
SLAC	-	0.1	-	937

2 Supplementary Figure

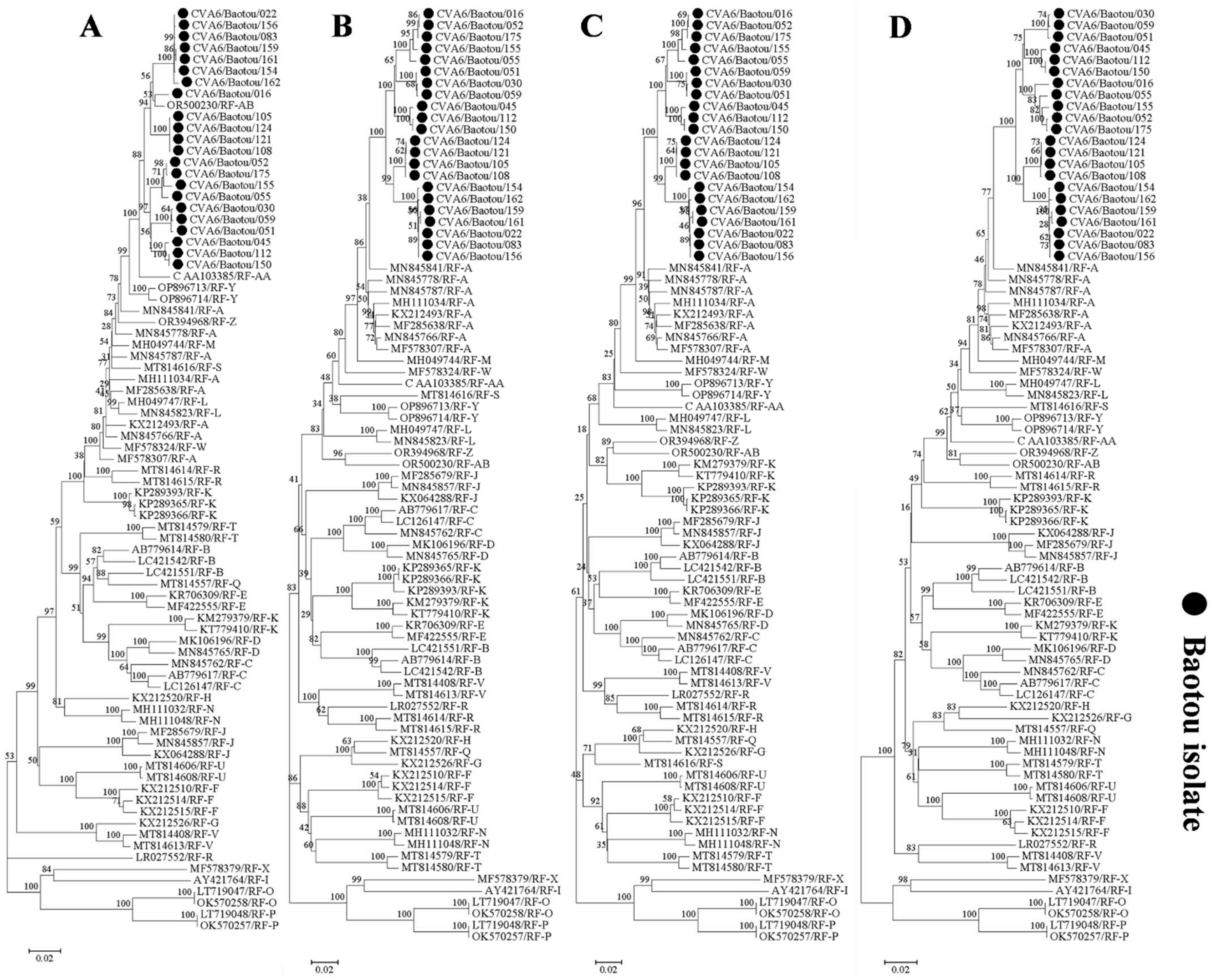

Figure S1. Phylogenetic analysis using the maximum-likelihood method with 1000 bootstrap replicates was performed on the non-capsid protein regions of Baotou isolates (removing duplicates of identical sequences): (A) P2 region; (B) P3 region; (C) 3C-3D region; (D) P2-P3 region.

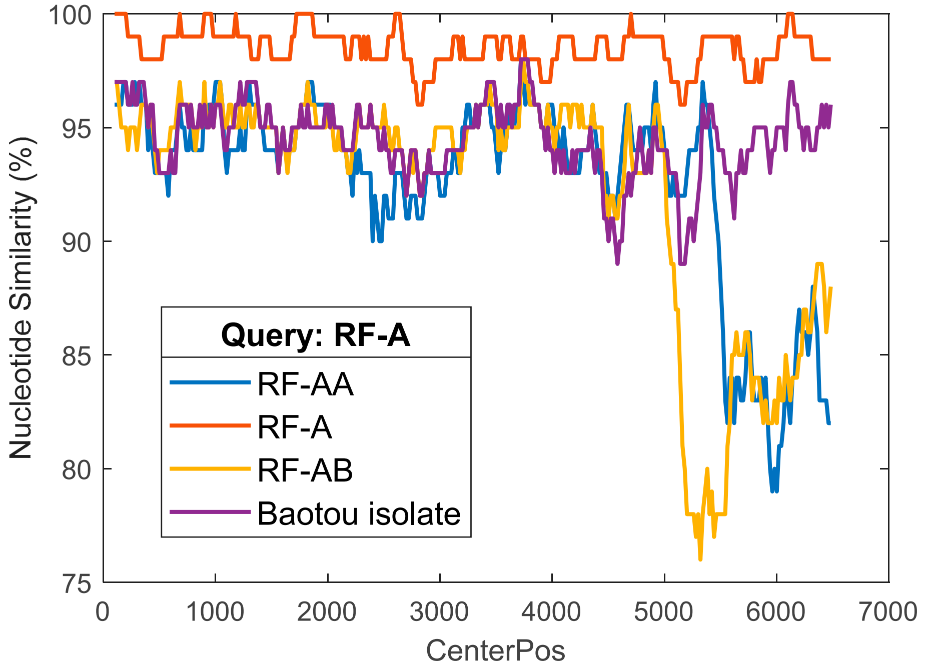

Figure S2. Similarity analysis of Baotou isolates was performed using a sliding window of 200 NT with a step size of 20 NT.
